# The Comparative Analysis of Buccal Exfoliated Cells in the Pediatric and Adolescent Age Groups Among the Dravidian Population During the COVID-19 Pandemic: A Cross-Sectional Study

**DOI:** 10.7759/cureus.44022

**Published:** 2023-08-24

**Authors:** Priyadharshini G, Pratibha Ramani

**Affiliations:** 1 Oral and Maxillofacial Pathology, Saveetha Dental College, Chennai, IND; 2 Oral Pathology and Microbiology, Saveetha Dental College and Hospitals, Saveetha Institute of Medical and Technical Sciences, Saveetha University, Chennai, IND

**Keywords:** covid-19, adolescent, pediatric, smear, cytology

## Abstract

Introduction

Adolescence is a distinct stage of development marked by a variety of physiological, behavioral, hormonal, and cellular changes.

Objective

The objective of this study is to assess the cytomorphometry of buccal exfoliated cells in the age groups 1-10 and 11-19 and detect any changes in their buccal exfoliated cells during the coronavirus disease 2019 (COVID-19) pandemic.

Materials and methods

Buccal smears were collected from 60 patients of age groups 1-10 (n=30) and 11-19 (n=30). Cytomorphometric analysis was done using the ImageJ software (National Institutes of Health, Bethesda, MD). Statistical analysis was done using an independent t-test.

Results

The cytomorphometric analysis of buccal smears between age groups 1-10 and 11-19 showed a statistically significant difference in cell size, cell shape, and nuclear-cytoplasmic (N/C) ratio.

Conclusion

This study helps to understand the physiological changes in this era and thus will aid to compare the physiological to the pathological changes of exfoliated cells in future studies.

## Introduction

Exfoliative cytology is the microscopical assessment of shed or desquamated cells from the epithelial surface, which is a rapid, painless, and less time-consuming procedure [1]. Being a noninvasive technique, it is easily accepted by the patients and therefore serves as a relatively inexpensive and easily accessible procedure to assess the physiological changes in normal individuals and helps us to give a thorough understanding of the normal physiology of the body [2]. It can also be used in cases where surgery is contraindicated such as in systemically compromised patients and patients with bleeding disorders. It is also valuable in mass screening [1]. For diagnostic, follow-up, and research purposes, the exfoliative cytology method can be performed several times.

Epithelial physiology is the foundation of exfoliative cytology. The loss of cell surface occurs on a regular basis in normal epithelium, and the thickness of the epithelium remains constant. Under normal conditions, epithelial cells are firmly fixed in place. The cells lose their cohesive force and exfoliate in the presence of any underlying pathology. As the cell cohesiveness is lost, exfoliated cells can be collected and examined under a microscope [3].

The oral cavity can be thought of as the perfect location to observe the effects of aging. The oral epithelium is continuously replaced by undergoing periodic turnover, indicating that this tissue should offer measurable signs of aging. The buccal mucosa especially serves as a promising site for smear collection as it enables quick and noninvasive smears [4]. Buccal cells can be thought of as the boundary between the body's internal and external environments. Therefore, changes in the buccal epithelial cell's functional activity mostly reflect the body's condition of local and systemic homeostasis or its impairment with aging [1]. As age progresses, the renewal capacity of tissues declines to show age‐related changes irrespective of gender [5]. There can also be various environmental effects on the cytomorphometric changes in children and young adults [6].

In addition to this, there are various hormonal influences, especially in the prepubertal and pubertal age groups, which show changes in the cytodifferentiation of the epithelium. Many tissues and epithelia undergo maturational changes as a result of estrogens and other steroid hormones. It is known that these hormonal stimuli generate distinct changes in the morphology of the epithelium in the buccal and vaginal mucosae. The degree of cell maturation increases with the number of superficial cells in the epithelium [7]. It has been proved in vaginal smears that the degree of maturation of exfoliated cells is highly influenced by hormonal changes. The appearance of more superficial cells may be attributed to the influence of the estrogen hormone, whereas progesterone influences the appearance of more intermediate cells [8]. Thus, cytology can help determine age-related changes and gender-related changes by helping to visualize cellular shape and size in the exfoliated cells [9].

Coronavirus disease 2019 (COVID-19) was declared a public health emergency and a global pandemic, which had a variety of psychosocial effects on people at the individual, national, and international levels [10]. The influence of factors that affect the timing and pace of puberty, such as obesity and psychological health, may have been amplified by the lockdown [11,12]. The developmental window throughout infancy, early childhood, or even adolescence is vulnerable, in which the occurrence of any stressful events during crucial times can cause short- and long-term physiological, cognitive, and behavioral consequences [11].

Despite the fact that several quantitative cytomorphologic and cytomorphometric research on pathological changes present in the oral cavity have been conducted, very few investigations have been made on the clinically normal oral mucosa to evaluate the physiological age- and gender-related changes under environmental influences. Studies that reported on physiological alterations, especially in the buccal mucosa, pertaining to the pediatric age group in the COVID-19 era are also very little [13]. The majority of studies pertaining to physiological age-related changes have compared the changes in smears between the young age group and the old age group. The pediatric age group should be a very important age group of the target as understanding their normal physiology helps to detect any pathological deviation. Because of this, it was felt judicious to carry out a study on further age-related changes in clinically normal oral squames between the pediatric age group of ages 1-10 and adolescents of ages 11-19 during the COVID-19 era. Thus, the aim of our study was to assess the cytomorphology of buccal exfoliated cells in the age groups 1-10 and 11-19 and detect any changes in their buccal exfoliated cells during the COVID-19 pandemic.

## Materials and methods

The study was a cross-sectional study done at Saveetha Dental College, Chennai, Tamil Nadu, between December 2021 and February 2022 after obtaining ethical committee clearance (Saveetha Dental College) (ethical approval number: IHEC/SDC/OPATH-2101/22/660). The study was ethically conducted in accordance with the Declaration of Helsinki. The study subjects were randomly selected from the patients reporting to the outpatient department of our institution. The sample size was calculated using the G*Power software (Universität Düsseldorf, Düsseldorf, Germany), based on the article by Nagarathinam et al. [14]. Patients with clinically normal buccal mucosa between the ages of one and 19 were included in the study, regardless of gender, socioeconomic status, or clinical status. Patients with intraoral swelling and other oral lesions such as ulcers were excluded. For each case, a prestructured pro forma was employed to gather pertinent data from the patients, which included age, gender, chief complaint, child habits, and dental status of the patient. Informed consent was obtained from the parents of the study participants. The sample size was 60 patients of which 30 were of the age group 1-10 and 30 were of the age group 11-19. Out of the total 60 participants, 31 were males, and 29 were females. For smear collection, patients were asked to rinse their mouths with water to remove any debris prior to smear collection. Two smears were collected from the buccal mucosa of each group of patients. Smears were collected by gently scraping the buccal mucosa using a moist wooden spatula and immediately smeared over a glass slide. The second smear was also gathered from the same site. The smears were fixed using 95% ethyl alcohol for 30 minutes. The slides were stained using both Papanicolaou stain and hematoxylin and eosin (H&E) stain, and a microscopic examination of the cytosmears was done.

**Figure 1:**
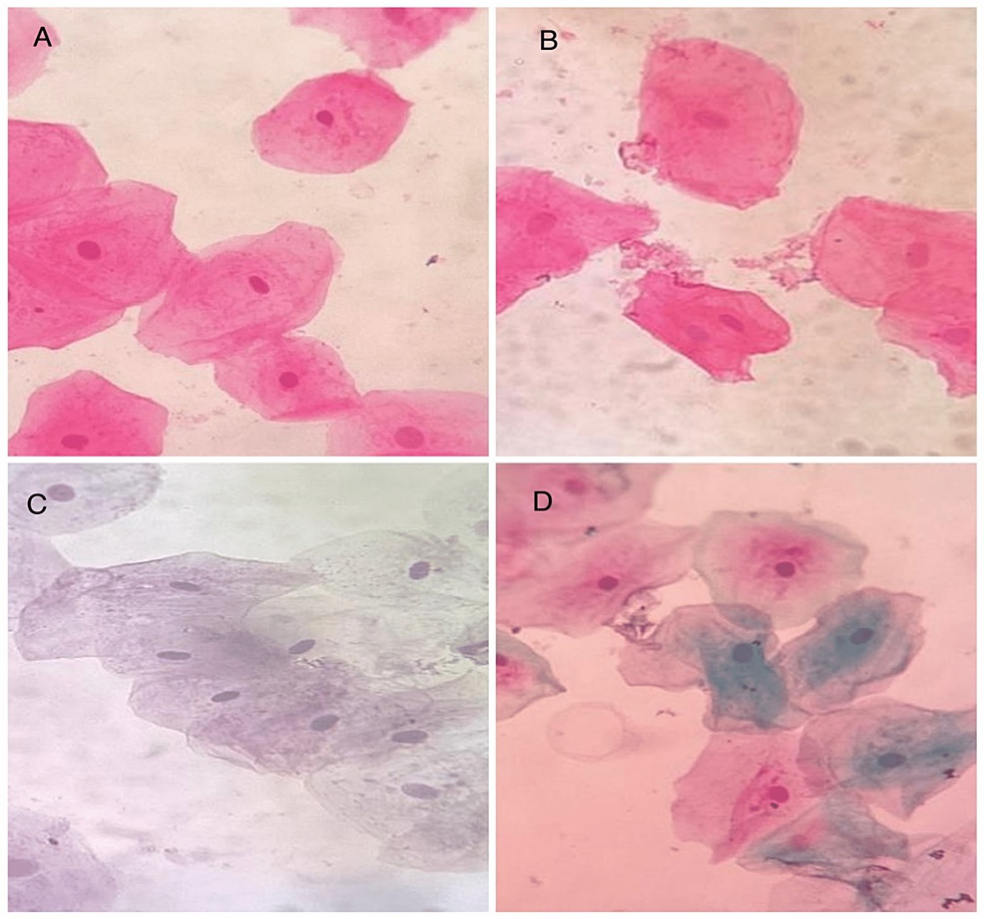
(A and B) H&E-stained buccal smears at 40× objective. (C and D) Pap-stained buccal smears at 40× objective H&E: hematoxylin and eosin

**Figure 2:**
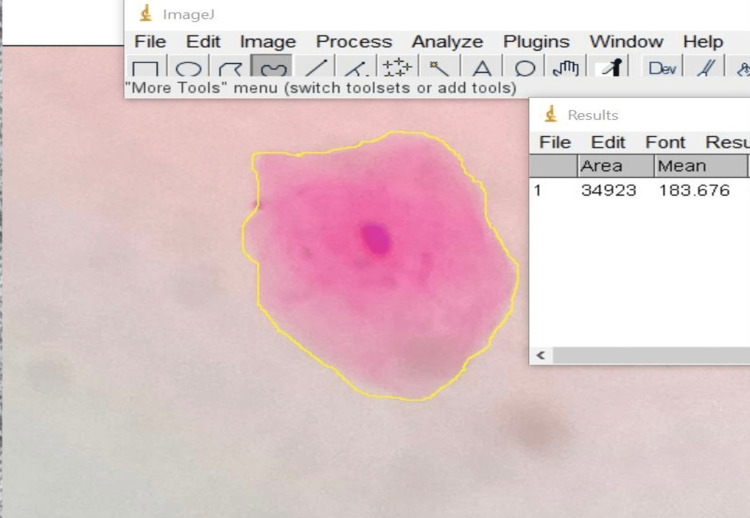
Cytomorphometric analysis using the ImageJ software

Photomicrographs were taken at both 10× and 40× magnification and were transferred to a computer (Figure 1). For image analysis, an average of 10 cells with defined outlines was selected per subject. Overlapping cells were not selected for image analysis. The software used for image analysis is the ImageJ software version 1.53 (National Institutes of Health, Bethesda, MD). In order to avoid errors in the measurement of cells, cells were measured stepwise from the top left corner to the right and then down. The cells were measured for their nuclear size, cytoplasmic size, and nuclear-cytoplasmic (N/C) ratio (Figure 2). The cellular size and nuclear size were determined after the cellular and nuclear outlines were outlined using a digitalized cursor and a computer-based measurement tool in the software. The cytoplasmic size was calculated as the sum of the differences between cellular size and nuclear size. The formula NC=nuclear size/cytoplasmic size was used to determine the nuclear-cytoplasmic ratio.

Statistical analysis was carried out using the Statistical Package for Social Sciences (SPSS) version 21 (IBM SPSS Statistics, Armonk, NY). The difference in cell size, nuclear size, and nuclear-cytoplasmic ratio between the age groups 1-10 and 11-19 was calculated using Student's t-test. These parameters were also calculated and compared between males and females in the age group between one and 19 using Student's t-test, and a p-value of <0.05 was considered significant.

## Results

The cytomorphometric analysis of buccal smears between age groups 1-10 and 11-19 showed a statistically significant increase in cell size (p=0.02) in the age group 11-19 compared to the age group 1-10 with a mean cellular value of 22.75±6.6 and 27.78±9.5, respectively.

**Figure 3:**
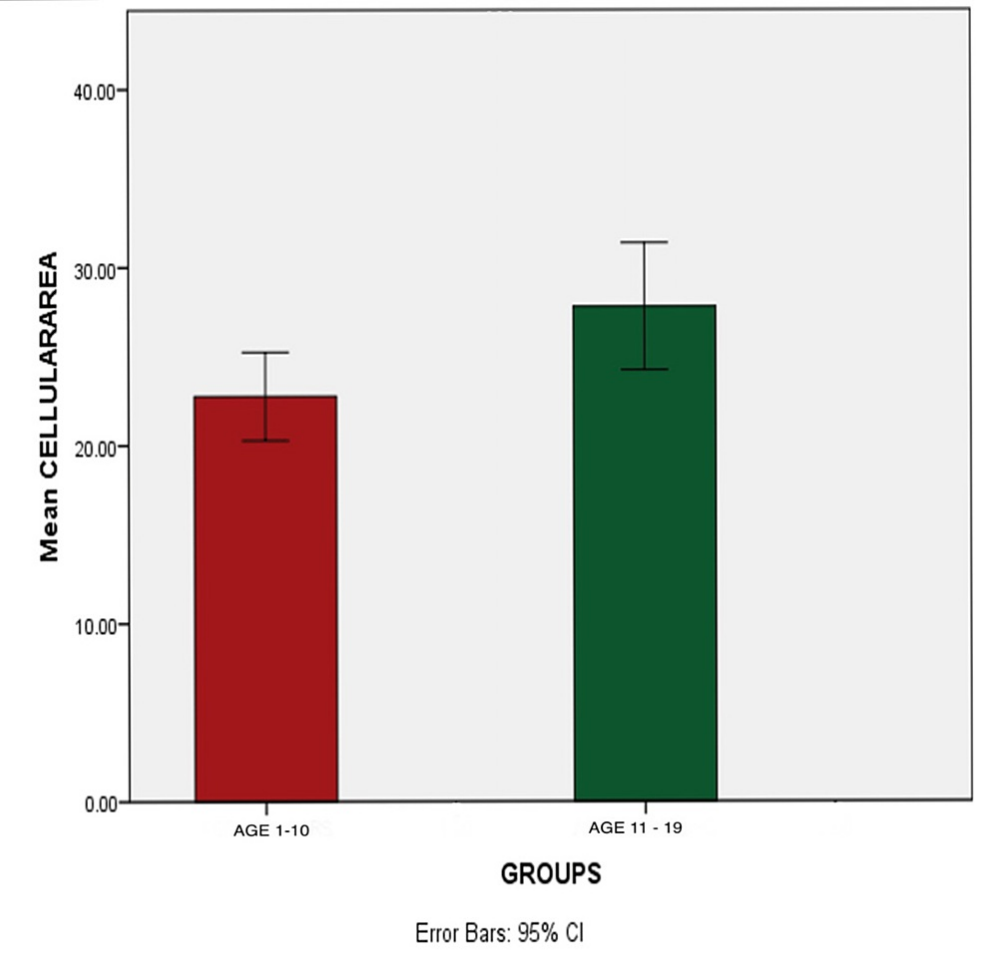
Comparison of cell size between the age groups 1-10 and 11-19

**Figure 4:**
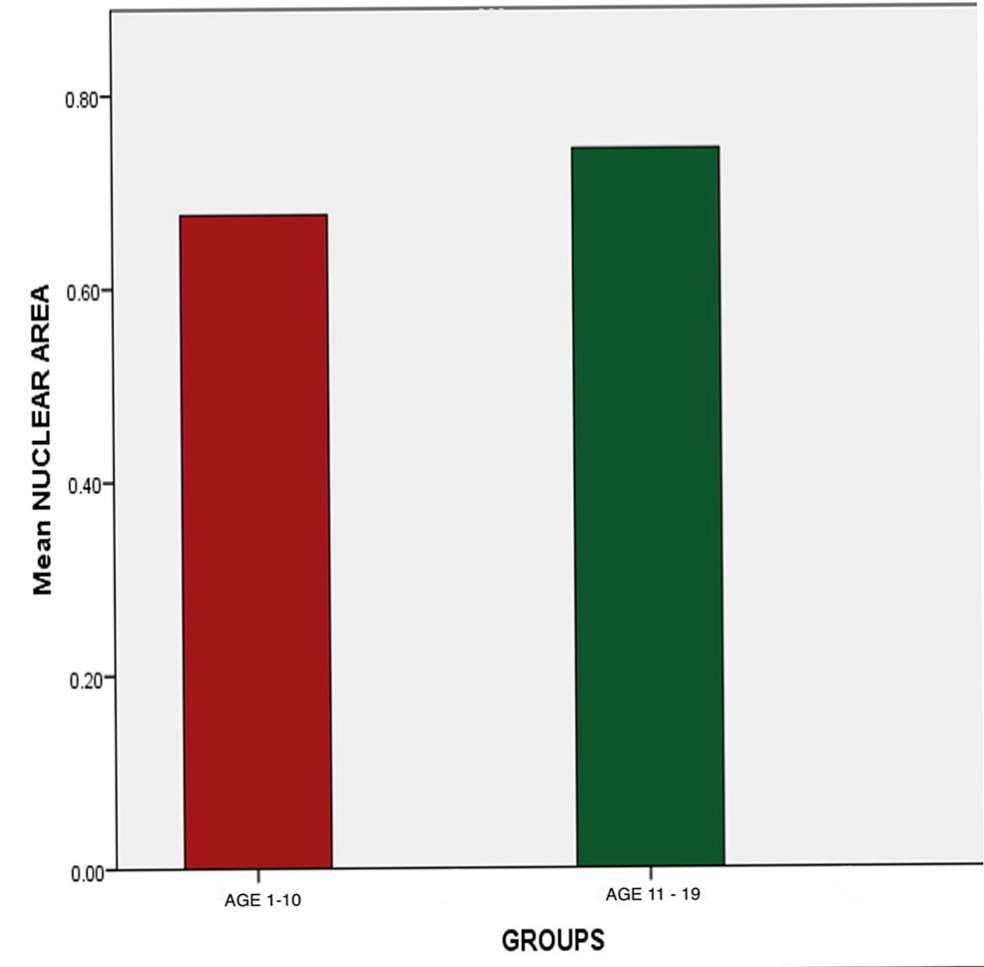
Comparison of nuclear size between the age groups 1-10 and 11-19

**Figure 5:**
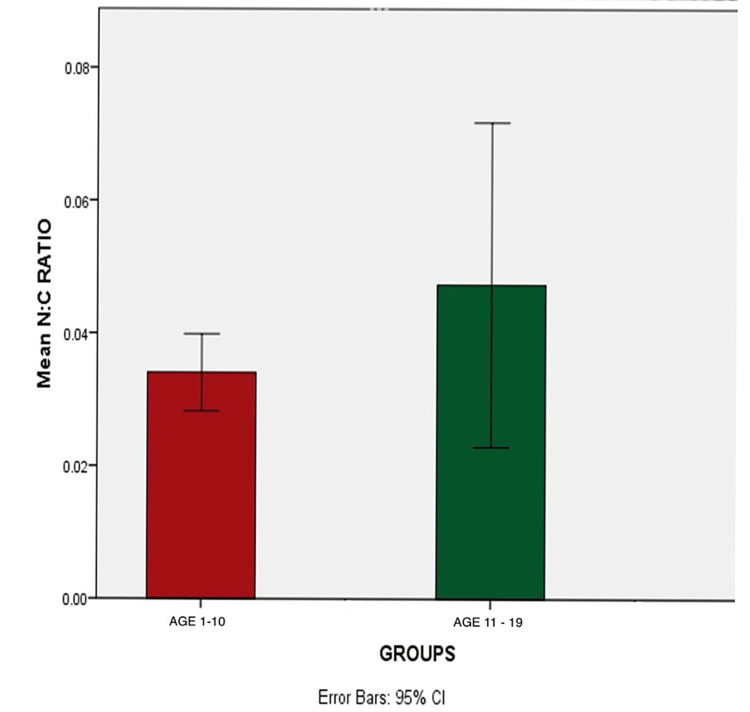
Comparison of nuclear-cytoplasmic (N/C) ratio between the age groups 1-10 and 11-19

There is an increase in nuclear size in the age group 11-19 compared to the age group 1-10 (p=0.230). The increase in nuclear-cytoplasmic ratio in the age group 11-19 was also noted (p=0.285) (Tables 1, 2 and Figures 3-5).

**Table 1:** Cell size, nuclear size, and nuclear-cytoplasmic (N/C) ratio of the age groups 1-10 and 11-19

Groups	Cell size (mean±standard deviation)	Nuclear size (mean±standard deviation)	N/C ratio (mean±standard deviation)
Ages 1-10	22.75±6.6	0.67±0.18	0.03±0.01
Ages 11-19	27.78±9.5	0.74±0.24	0.04±0.06

**Table 2:** Comparison of cell size, nuclear size, and nuclear-cytoplasmic ratio between the age groups 1-10 and 11-19 by independent t-test SD: standard deviation

Groups	Mean±SD	p-value
Cell size in ages 1-10 and 11-19	5.03±0.54	0.02
Nuclear size in ages 1-10 and 11-19	0.07±0.06	0.23
Nuclear-cytoplasmic ratio in ages 1-10 and 11-19	0.001±0.05	0.285

**Figure 6:**
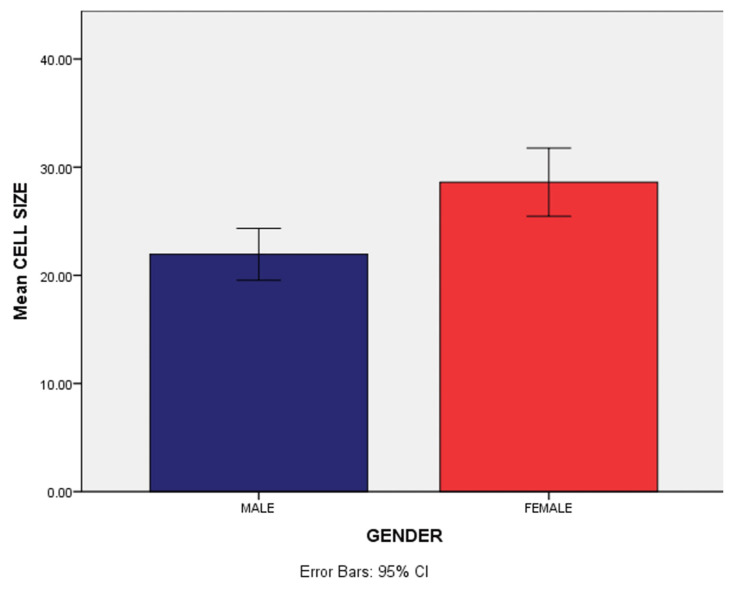
Comparison of cell size between males and females of the age group 1-19

**Figure 7:**
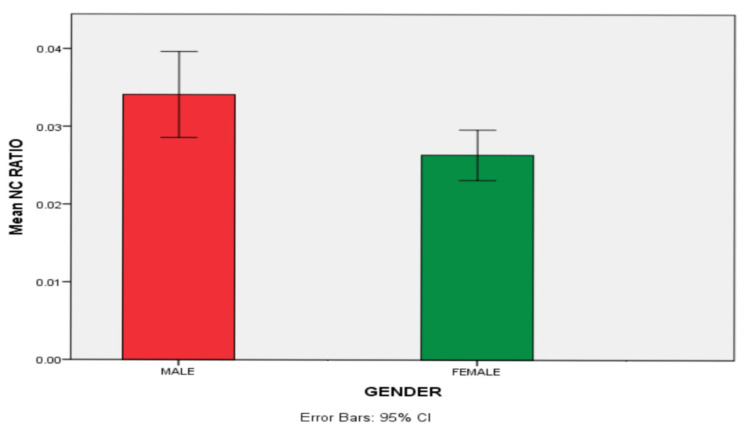
Comparison of mean nuclear-cytoplasmic (N/C) ratio between males and females of the age group 1-19

There is also a significant increase in the cell size of females when compared between the male and female populations of the age group 1-19 (p=0.01). There was an increase in nuclear size (p=0.5) in females compared to males, but it was not statistically significant. There was also a statistically significant increase in nuclear-cytoplasmic ratio (p=0.01) in males compared to females (Tables 3, 4 and Figures 6, 7).

**Table 3:** Cell size, nuclear size, and nuclear-cytoplasmic (N/C) ratio between males and females of ages 1-10 and 11-19

Groups	Cell size	Nuclear size	N/C ratio
Male	21.94±6.4	0.69±0.18	0.03±0.01
Female	28.61±8.4	0.73±0.24	0.02±0.008

**Table 4:** Comparison of cell size, nuclear size, and nuclear-cytoplasmic ratio between males and females between the age groups 1-10 and 11-19 by independent t-test SD: standard deviation

Groups	Mean±SD	p-value
Cell size between males and females	6.67±1.98	0.01
Nuclear size between males and females	0.04±0.06	0.05
Nuclear-cytoplasmic ratio between males and females	0.01±0.092	0.01

## Discussion

Adolescence is a developmental stage between childhood and adulthood that is characterized by numerous behavioral, hormonal, and cellular changes. The second decade of life, or adolescence as the World Health Organization (WHO) defines it (10-19 years old), is a period of profound physical, psychological, and social transformation. In our study, there is a significant difference in cell size (ages 1-10, 22.75; ages 11-19, 27.78) (p=0.021) between the age groups 1-10 and 11-19, which was consistent with the study conducted by Donald et al. [13], in which there was a significant change in cell size between the age groups 5-15 and 15-30 in apparently healthy individuals. This could be attributed to the change in testosterone and estrogen levels in their blood, especially during the pubertal phase, which causes the acceleration of cell metabolism and growth in young adults. Larger cell size in young adolescents could be attributed to an increased and active cell cycle, active mitosis, and active function that leads to increased renewal and regeneration [13].

In a study conducted by Shetty et al. [9], there was a significant decrease in the average cell size of the pediatric and adult population (age groups 5-15 and 15-30 years). This may be attributed to various distinct signaling, transcriptional, and epigenetic variables in the pediatric age groups that are thought to function, resulting in a general state of development that promotes growth. During particular developmental windows, environmental exposures throughout development, or the so-called exposome, may have consequences on children's and adults' health and tissue function at the cellular level. It may be related to health disparities and cellular and tissue responses to poor socioeconomic status, stress, inflammation, and environmental pollution [9].

The difference in the nuclear-cytoplasmic (N/C) ratio (p=0.285) (ages 1-10, 0.03; ages 11-19, 0.04) was observed in our study, which was consistent with the results of the study conducted by Anuradha and Sivapathasundharam [15], wherein there was a difference in the nuclear-cytoplasmic ratio in different age groups. This may be due to cell mechanical behavior, which influences cell growth and cell maturation [16].

Patel et al. [16] have observed in normal gingival smears a significant difference in nuclear size, cell size, and nuclear-cytoplasmic ratio between males and females irrespective of age. This was consistent with the results obtained in our study wherein there was a significant difference in cell size between males and females of the age group 1-19. This could be attributed to age-related adolescent changes, which may be puberty-dependent because they depend on the increase in gonadal hormones throughout puberty. However, other alterations during this same general developmental period may be influenced by more widespread, puberty-independent ontogenetic processes [17].

In a study by Verzani et al. [18], they found that precocious puberty cases in females have increased significantly after the COVID-19 pandemic began, compared to the same six-month period in 2019. This may be related to the results obtained in our study with increased cell size in females. A sedentary lifestyle, the increased use of electronics, and a high intake of caloric food could have contributed to hormonal disturbances and in turn cellular changes in the adolescent age groups especially in females. There can also be the influence of various psychological factors such as stress, anxiety, and depression during the COVID-19 phase [18].

As there is a paradigm shift in cellular growth and development, as well as the onset of puberty in the past 10 years, especially in the COVID-19 era, there is a need to understand the physiological hormone-related changes in children in this era. Understanding the morphological changes of exfoliated cells in different age groups gives the basis for physiology and helps to assess the changes in normal morphology. Morphological changes in the oral exfoliated cells can be due to fluctuating levels of hormones such as growth hormone, estrogen, testosterone, and progesterone [9,17].

Epithelial cells show tight junctions, anchoring junctions, and gap junctions allowing varying degrees of cellular interactions. It helps in adhesion to extracellular matrix and influences cell shape. Intercellular communication alongside the transfer of ions and small molecules is also crucial in understanding the physiology of epithelial cells in homeostasis or in diseased states. Epithelial cells also undergo cell division and apoptosis, migration, and fluid pumping that modifies tension and cytoskeleton, which are ultimately governed by genetic regulation that reflects in cell morphology.

The importance of improving our understanding of normal morphology and its deviation beginning in early infancy cannot be overstated because many childhood-onset diseases progress to lifelong, chronic illnesses. Only after the fundamental observations in normal oral mucosal cells are established can the pathology be examined.

Furthermore, cytomorphometric analysis in the pediatric age groups can be done to assess hormone-related disorders such as dwarfism and acromegaly. Thus, this study can be considered a benchmark in assessing the normal growth phase in children, which enables us to identify any delay or accelerated growth pattern in children during the COVID-19 era.

As a future scope, studies must be carried out to assess if there are any changes in the cytomorphometry of buccal smears compared to the COVID-19 era, to establish whether lifestyle during the COVID-19 era had a significant impact on the physiological cellular growth pattern.

Limitations

The main limitation of the present study is that it has less sample size and was carried out in a single hospital-based setting. Clinical correlation has not been done, and a comparison of cellular changes between the COVID-19 pandemic and pre-COVID-19 pandemic has not been done.

## Conclusions

Our observation showed a difference in the morphology of buccal exfoliated cells between the age groups 1-10 and 11-19. This study will aid us in distinguishing between normal and abnormal morphology and ensuring the normal physiological increase in cellular and nuclear sizes and increased nuclear-cytoplasmic ratio in young adolescents, as well as the increase in all these parameters in females compared to that of the males of the same age group 1-19. These changes should not be misdiagnosed as a deviation from normal morphology. Thus, this may serve as a tool to assess hormone-related normal and abnormal changes, especially in the pubertal growth phase during the COVID-19 pandemic.
